# Genetic Evaluation and Selection of *Eucalyptus urophylla* Families and Hybrids for Cold Tolerance in Northern Guangdong, South China

**DOI:** 10.3390/plants15162505

**Published:** 2026-08-19

**Authors:** Min Zhu, Guolong Li, Yang Hu, Zhikang Hu, Yizhen Liu, Guohui Wu, Shouguang Chen, Zhiyong Liu, Jianmin Xu, Zhaohua Lu, Guangyou Li

**Affiliations:** 1 Key Laboratory of State Forestry and Grassland Administration on Tropical Forestry, Research Institute of Tropical Forestry, Chinese Academy of Forestry, Guangzhou 510520, China; 13639291253@163.com (M.Z.); 18394926956@163.com (G.L.); huzk@caf.ac.cn (Z.H.); zyliu_hina@163.com (Z.L.); jianmxu@163.com (J.X.); 2 College of Landscape Architecture, Nanjing Forestry University, Nanjing 210037, China; 3 Xinhui Research Institute of Forestry Science, Jiangmen 529100, China; huyangchong@126.com (Y.H.); 13536217116@163.com (S.C.); 4 Jiangmen Research Institute of Forestry Science, Jiangmen 529099, China; lks-jm@163.com; 5 Lianshan Forest Farm of Guangdong, Qingyuan 513200, China; 13729670940@163.com

**Keywords:** *Eucalyptus urophylla*, cold tolerance, heterosis, breeding value, SCA, GCA

## Abstract

To identify superior eucalyptus genotypes adapted to the cold-prone northern margin of Guangdong Province and to elucidate the genetic basis underlying growth, cold tolerance, and heterosis, this study evaluated 51 eucalyptus genetic materials with diverse genetic backgrounds, including *Eucalyptus urophylla* families and *E. urophylla*-derived interspecific hybrid combinations. Growth and cold tolerance traits were measured at 4.0 and 6.5 years after planting to systematically assess genetic variation, combining ability, and selection potential among different genetic materials. Significant genetic variation was observed among the tested materials for both growth and cold tolerance traits. Several families and hybrid combinations exhibited superior performance in stem volume production and cold tolerance, indicating substantial potential for genetic improvement. BLUP analysis revealed a genetic trade-off between growth and cold tolerance in 31 families; however, several elite genotypes exhibited a favorable combination of rapid growth and enhanced cold tolerance, making them promising candidates for future breeding programs. Combining ability analysis demonstrated that both additive and non-additive genetic effects contributed to growth performance, and superior parental combinations with high general combining ability and specific combining ability effects were identified. Furthermore, interspecific hybrid evaluation showed that *E. urophylla* × *Eucalyptus grandis* hybrids generally exhibited greater growth potential and more stable productivity, whereas *E. urophylla* × *Eucalyptus camaldulensis* hybrids displayed stronger adaptation to low-temperature stress. These findings provide valuable genetic resources and theoretical support for developing fast-growing, cold-tolerant eucalyptus cultivars and improving the stability and productivity of subtropical plantation forests.

## 1. Introduction

*Eucalyptus urophylla*, an economically important pioneer species in the family *Myrtaceae*, is widely planted because of its rapid growth, broad adaptability, short rotation cycle, and strong resistance to pests and diseases [1]. It is a major plantation species for timber and biomass production and provides an important raw material for the global pulp and paper industry [2]. In China, *E. urophylla* is extensively cultivated across the southern provinces, including Guangxi, Guangdong, and Hainan [3]. However, *E. urophylla* is naturally distributed in tropical regions such as Indonesia and has relatively low inherent cold tolerance. Low-temperature and frost damage therefore limit its expansion into higher-altitude and higher-latitude areas. Extensive eucalyptus introduction trials have shown that eucalyptus plantations are particularly vulnerable to freezing injury, and in recent years, frequent frost, freezing, and low-temperature events in southern China have further constrained eucalyptus cultivation and productivity in these regions [4,5,6]. Therefore, improving cold tolerance while maintaining rapid growth is a key objective in eucalyptus genetic improvement and is fundamental to expanding the suitable cultivation range and enhancing the stability and productivity of plantations.

Hybrid breeding is a widely adopted strategy for improving productivity, environmental adaptation, and stress tolerance in eucalyptus [7]. By exploiting heterosis, it combines favorable alleles from genetically diverse parents through controlled or open pollination, leading to improvements in growth, stem volume, wood quality, and resistance to environmental stresses [8,9,10,11,12]. Superior hybrids not only provide elite germplasm for cultivar development and commercial deployment but also serve as valuable parental resources for recurrent breeding and long-term genetic improvement [13,14].

General combining ability (GCA) and specific combining ability (SCA) are widely used to characterize additive and non-additive genetic effects, respectively, and provide complementary information for evaluating parental performance and hybrid combinations [15,16]. Together with best linear unbiased prediction (BLUP) of breeding values and heterosis analysis, these approaches enable a comprehensive assessment of genetic potential, facilitating the identification of elite parents and superior families while improving understanding of the genetic basis of growth and cold tolerance [17,18,19]. However, existing studies largely focus on individual genetic parameters or early growth performance, and comprehensive evaluations of heterosis across stand ages and its underlying genetic mechanisms remain limited, reducing the transferability of breeding strategies across populations and environments [20]. In addition, growth and stress tolerance are commonly linked by a resource allocation trade-off, whereby enhanced growth is often associated with reduced stress resistance [21]. Whether this trade-off is universal in *Eucalyptus* hybrid populations, and whether coordinated improvement of growth and cold tolerance can be achieved through optimized parental selection and genetic recombination, remains unclear.

Based on these knowledge gaps, we hypothesize that *E. urophylla* hybrid progeny exhibit substantial genetic variation in growth and cold tolerance, and that appropriate parental combinations can simultaneously improve both traits. To test this hypothesis, we evaluate a hybrid population established in the cold-prone northern margin of Guangdong Province using measurements collected at 4.0 and 6.5 years. Growth performance, cold tolerance, and survival are assessed by integrating BLUP-based breeding values, heterosis, general combining ability (GCA), and specific combining ability (SCA). Specifically, this study aims to (i) identify superior families combining rapid growth with enhanced cold tolerance across stand ages, (ii) evaluate the persistence of heterosis and its relationship with cold tolerance, and (iii) quantify the relative contributions of additive and non-additive genetic effects to stem volume in order to identify elite parents and superior hybrid combinations. The findings provide new insights into the genetic basis of growth and cold tolerance in eucalyptus hybrids and support the development of efficient breeding strategies for cold-tolerant eucalyptus in subtropical regions.

## 2. Results

### 2.1. Growth Performance and Analysis of Variance at 4 and 6.5 Years

**Figure 1:**
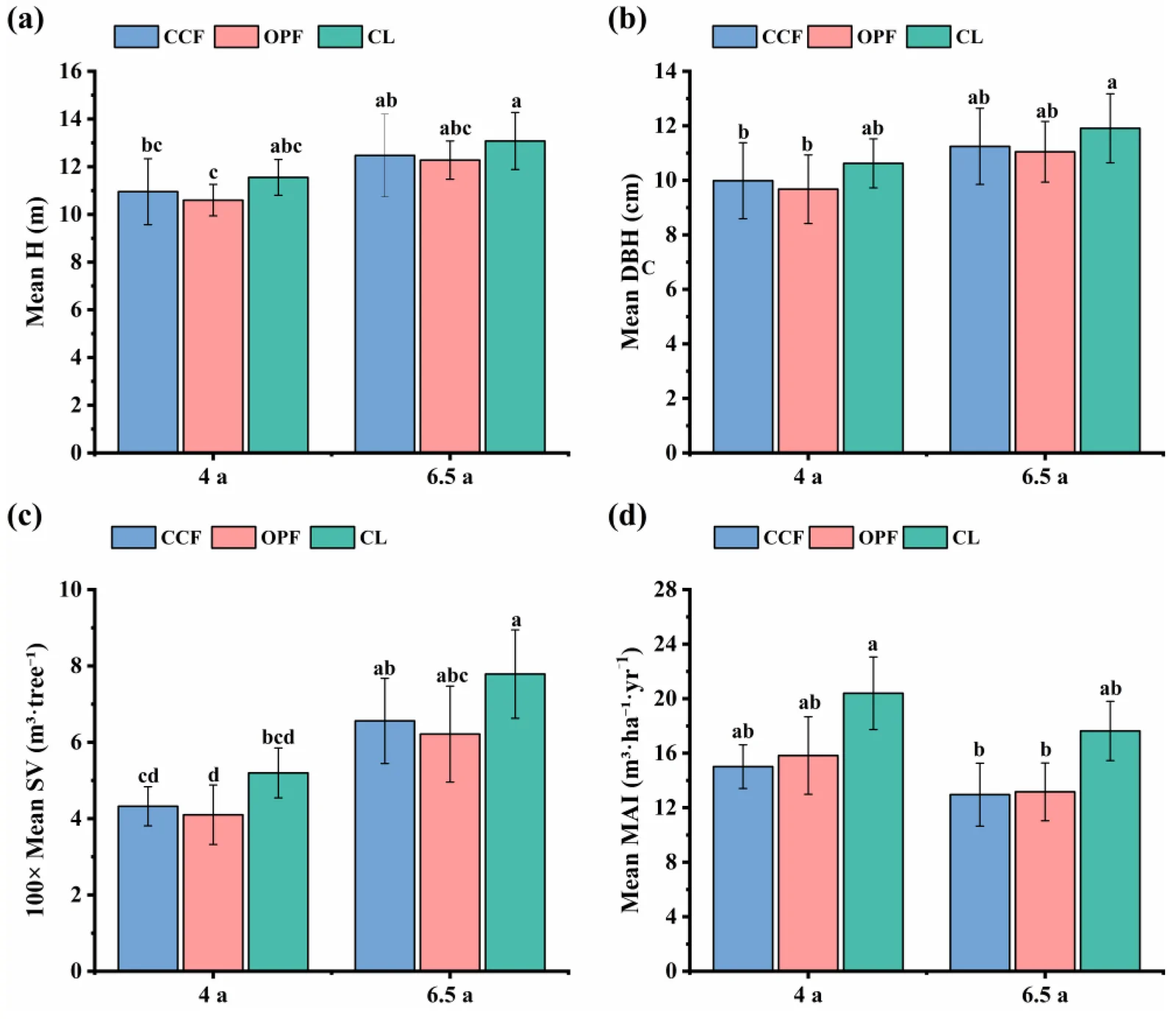
Comparison of mean growth traits among different genetic materials at two stand ages (4.0 and 6.5 years). (**a**) Tree height (*H*); (**b**) diameter at breast height (*DBH*); (**c**) 100× individual stem volume (100× *SV*); (**d**) mean annual increment (*MAI*). Note: CCF, controlled-cross families; OPF, open-pollinated families; CL, clonal controls. Different lowercase letters in panels (**a**–**d**) indicate significant differences among genetic material types (*p* < 0.05).

The mean values of tree height (*H*), diameter at breast height (*DBH*), stem volume per tree (100× *SV*), and mean annual increment *(MAI*) are compared among different genetic materials at 4.0 and 6.5 years (Figure 1). Stem volume per tree is substantially greater at 6.5 years than at 4.0 years, whereas *MAI* declines with stand age. These results indicate an age-related shift from rapid early growth to a slower but sustained phase of biomass and stem development.

The mixed-effects model analysis (Table 1) showed significant differences among genetic materials in growth traits at both 4 and 6.5 years of age. For the controlled-pollinated families (CCF, 40 families), significant to highly significant differences among families were observed for all three traits at both ages (4 years: F = 4.94–5.89, *p* < 0.001; 6.5 years: F = 2.34–2.85, *p* < 0.05), indicating substantial variation in growth performance among the CCF families. The open-pollinated families (OPF) showed strong family effects at both ages, with F-values generally higher than those observed in the CCF group (4 years: F = 5.06–7.55, *p* < 0.001; 6.5 years: F = 6.87–8.16, *p* < 0.001), suggesting greater genetic variation among the OPF families. In the clonal controls (CL, 4 clones), DBH and SV showed significant family effects at both ages (*p* < 0.05), whereas tree height did not differ significantly among clones at 4 years (*p* = 0.0761). Overall, the genetic materials exhibited varying degrees of between-family variation in growth traits, with CCF showing the most consistent family effects across ages, indicating good potential for family-level selection.

### 2.2. Multiple Comparisons of Growth and Cold Tolerance Traits Among Genetic Materials

**Figure 2:**
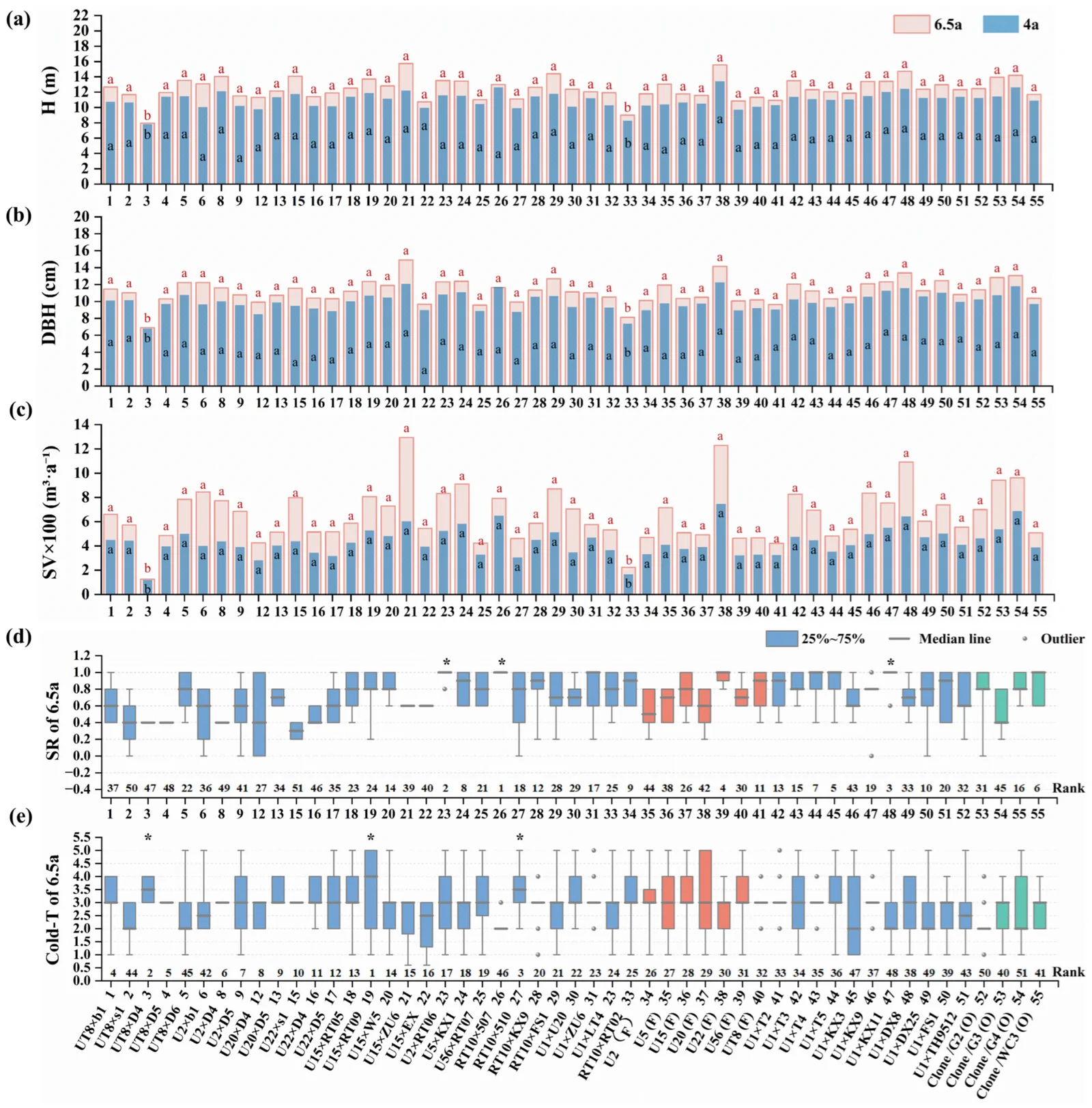
Multiple comparisons of major growth and cold tolerance traits among 51 genetic materials. (**a**) Tree height (*H*); (**b**) diameter at breast height (*DBH*); (**c**) stem volume per tree (*SV*) × 100; (**d**) survival rate (*SR*) at 6.5 years; and (**e**) cold tolerance (*Cold-T*) coefficient at 6.5 years. Note: Controlled-cross families (CCF, 1–33 and 41–51), open-pollinated families (OPF, 34–40), and control clones (CL, 52–55). In panels (**a**–**c**), different lowercase letters indicate significant differences among different genetic material types (*p* < 0.05). The numbers on the x-axes in panels (**d**,**e**) indicate the rankings of the genetic materials, and asterisks (*) indicate the top three ranked genetic materials. Different colors represent different genetic material groups: blue represents CCF, red represents OPF, and green represents CL.

Figure 2 illustrates variation in major growth and cold tolerance traits among different genetic materials at 4.0 and 6.5 years. Overall, tree height (*H*), diameter at breast height (*DBH*), and stem volume per tree (100× *SV*) increase substantially with stand age across all materials, although the magnitude of increase varies among families. Among the open-pollinated families (OPF), Family 38 (UF) consistently exhibits superior performance across *H*, *DBH* and *SV* at both stand ages. Among the 40 controlled-pollination families (CCF), 21 (U × U) and 48 (U × G) exhibited overall superior performance. In contrast, Families 3 (U × D) and 33 (C × C) show significantly lower values for *H*, *DBH*, and *SV* at both ages (*p* < 0.05).

For cold-related traits (Figure 2d,e), among the 40 controlled-cross families (CCFs), families 23 (U × C), 26 (C × P) and 33 (C × C) exhibited relatively high survival rates, whereas family 2 (U × S) had a significantly lower survival rate than the other materials (*p* < 0.05). For cold tolerance, families 19 (U × C), 27 (C × P) and 1 (U × B) showed relatively strong cold tolerance, whereas clone 52 (Clone) exhibited significantly weaker cold tolerance (*p* < 0.05). Compared with the other hybrid families, families 19, 21, 23, 24 and 29 showed both superior growth performance and relatively high cold tolerance, indicating overall superior performance. Notably, some materials with excellent growth performance, such as clone 52 (Clone), families 5 (U × D) and 2 (U × S), exhibited relatively weak cold tolerance, suggesting that a certain degree of genetic trade-off may exist between growth traits and cold tolerance.

### 2.3. Comprehensive Evaluation of Breeding Values Among Genetic Materials

**Figure 3:**
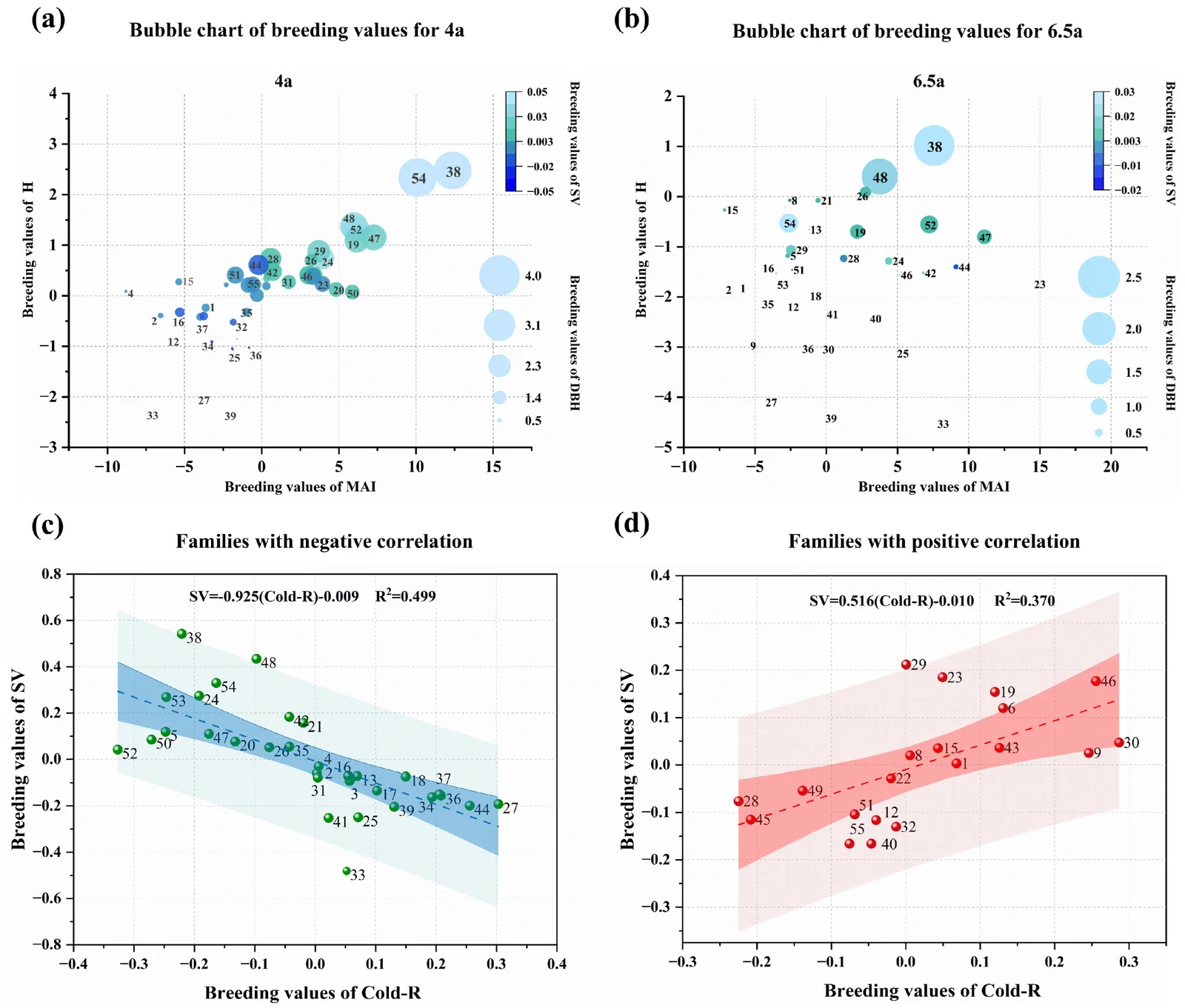
Breeding value evaluation across different genetic materials. (**a**) Bubble plot of integrated breeding values at 4 years of age; (**b**) bubble plot of integrated breeding values at 6.5 years of age. In the bubble plots, the *x*-axis represents the breeding value for mean annual increment (*MAI*), the *y*-axis represents the breeding value for tree height (*H*), bubble size represents the predicted breeding value for diameter at breast height (*DBH*), and color intensity represents the predicted breeding value for stem volume per tree (*SV*). (**c**) Families showing a negative correlation between breeding values for *SV* and cold tolerance; (**d**) families showing a positive correlation between breeding values for *SV* and cold tolerance. Note: In panels (**c**,**d**), the dashed lines represent the fitted linear regression lines, and the shaded areas represent the corresponding 95% confidence intervals (CIs). Complete breeding value data are provided in Supplementary Table S1.

Breeding values (*BV*) of parental materials are estimated using the best linear unbiased prediction (BLUP) method. As shown in Figure 3a,b, families with high overall breeding values at 4.0 years include 38 (UN), 54 (Clone), 48 (U × G), 52 (Clone), 47 (U × G), 19 (U × C), and 29 (C × G). Among them, Family 38 (UN, *BV_SV* = 0.0300) and Family 54 (Clone, *BV_SV* = 0.0302) show the highest stem volume breeding values, both exceeding 0.03. Within the controlled-pollinated families, relatively high values are observed in Families 48 (U × G, *BV_SV* = 0.0177), 24 (*BV_SV* = 0.0182), and 29 (*BV_SV* = 0.0105). At 6.5 years, the top-ranking materials include Families 38, 54, 23, 46, 19, 48, and 47. The number of families with stem volume breeding values exceeding 0.03 increases to three, namely 38 (UF, *BV_SV* = 0.0542), 48 (U × G, *BV_SV* = 0.0434), and 54 (Clone, *BV_SV* = 0.0330).

In terms of cold tolerance (Figure 3c,d), the breeding values for *Cold-T* at 6.5 years ranged from −0.3268 to 0.3031. Seven families exhibited relatively high cold tolerance breeding values: Family 27 (C × P; *BV_Cold-T* = 0.3031), Family 30 (U × U; 0.2868), Family 46 (U × G; 0.2556), Family 44 (U × T; 0.2556), Family 9 (U × D; 0.2461), Family 36 (UN; 0.2081), and Family 37 (UN; 0.2054). Correlation analysis showed that the breeding values for stem volume (*SV*) and cold tolerance were negatively correlated in 31 families but positively correlated in 20 families. Notably, the three families with the highest *SV* breeding values (Families 38, 48, and 54) had negative or near-zero breeding values for cold tolerance, suggesting a potential genetic trade-off between growth potential and cold tolerance.

Against this background, Families 46 (U × G; *BV_SV* = 0.0177, *BV_Cold-T* = 0.256), 19 (U × C; 0.0154, 0.120), 23 (U × C; 0.0185, 0.050), and 29 (C × G; 0.0212, 0.001) exhibited a relatively balanced performance in terms of growth and cold tolerance. In particular, Families 19 and 23 maintained *SV* breeding values above 0.01 at both stand ages, indicating high overall genetic potential. These families could therefore be retained as elite families and further evaluated as candidate breeding parents combining rapid growth with enhanced cold tolerance.

### 2.4. Heterosis Analysis of Stem Volume Among Genetic Materials

**Figure 4:**
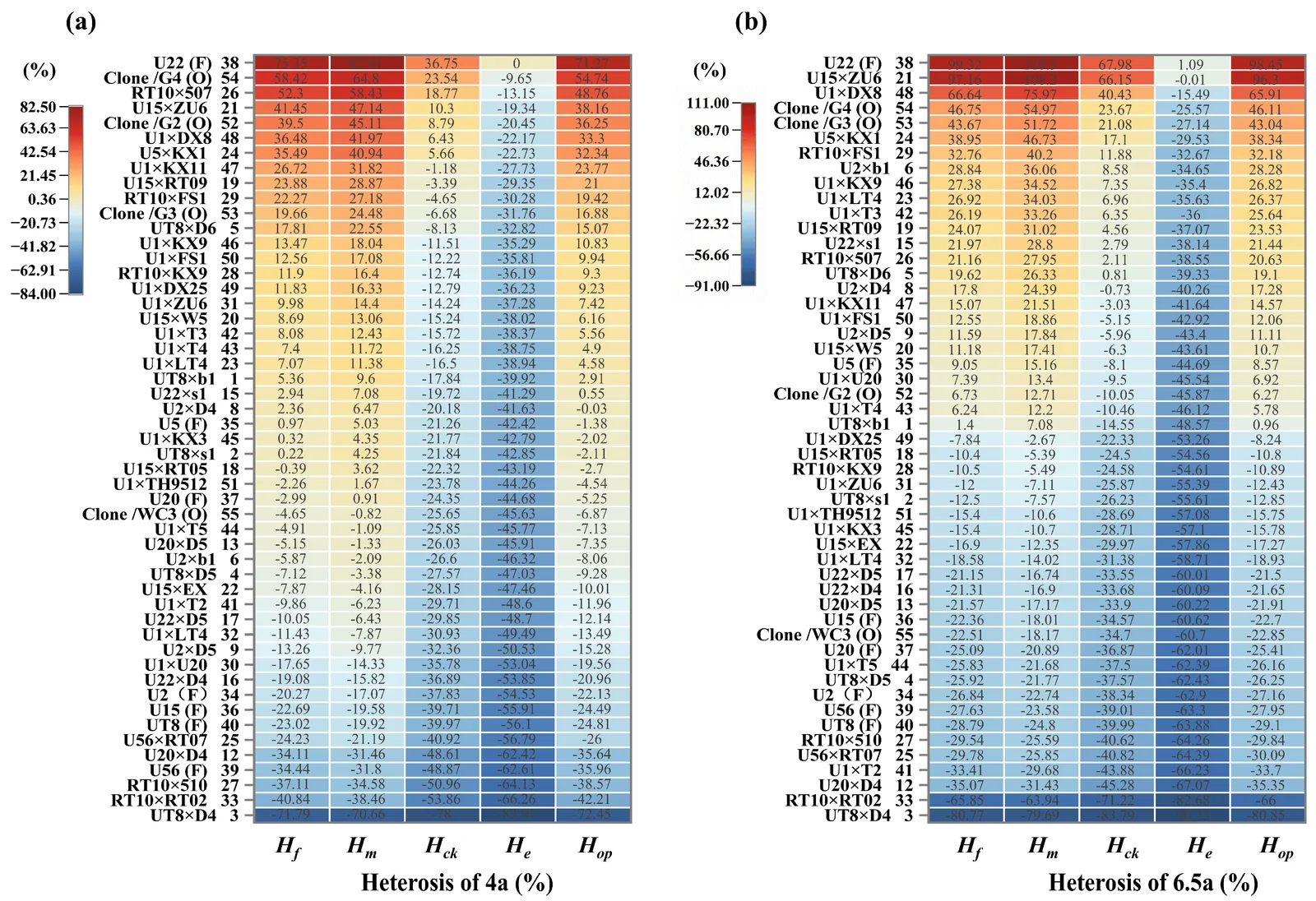
Heatmaps of stem volume heterosis across different genetic materials at 4.0 and 6.5 years. (**a**) 4.0 years; (**b**) 6.5 years. *H_op_*, over-population heterosis; *H_e_*, over-standard heterosis; *H_ck_*, over-clone heterosis; *H_m_*, over-natural open-pollinated heterosis; *H_f_*, Over-controlled open-pollinated heterosis. Positive values indicate higher performance than the corresponding reference group. Families are ranked by a composite index.

Stem volume heterosis is evaluated using five reference criteria (Figure 4). For mid-parent heterosis (*H_op_*), 23 families (45%) show positive values at 4.0 years, increasing to 25 families (49%) at 6.5 years. Three controlled-pollinated families (21, 48, and 24) consistently exhibit H*_op_* values above 30% at both stand ages, with Family 21 showing the highest increase (38.16% to 96.30%).

For standard heterosis (*H_e_*), using Family 21 at 6.5 years as the reference, only Family 38 shows a positive value (+1.09%), whereas all other families show negative values ranging from −0.01% to −90.25%. Families 21 and 38 exhibit similar stem volume levels, differing by approximately 1%. For clonal heterosis (*H_ck_*), only seven materials show positive values at 4.0 years, including four controlled-pollinated families (26, 21, 48, and 24). At 6.5 years, this number increases to 15, of which 12 are controlled-pollinated families. Families 21, 48, 24, and 29 exhibit *H_ck_* values exceeding 10% at 6.5 years.

For *H_m_*, the proportion of positive families decreases from 59% at 4.0 years to 49% at 6.5 years. Families 38 and 21 show more than twofold higher stem volume than the open-pollinated mean, with *H_m_* values of 110.50% and 108.20%, respectively. For *H_f_*, the mean heterosis decreases from 2.38% to 1.13% across the two stand ages.

Overall, heterosis patterns remain stable or slightly decline with stand age. Families 21, 48, and 24 show consistent performance across ages, with Family 21 exhibiting the highest mid-parent heterosis at 6.5 years *(H_op_* = 96.30%), together with Family 38 representing the highest-performing materials for stem volume.

### 2.5. Combining Ability Analysis of Stem Volume Among Different Genetic Materials

The GCA estimate (ĝi) represents the deviation of parental performance from the population mean, while the relative GCA effect (%) reflects the contribution of each parent to progeny stem volume performance relative to the population mean. As shown in Table 2, DX8 and KX1 were identified as male parents with the highest positive GCA effects, indicating superior additive genetic potential for stem volume improvement. Among female parents of *E. urophylla*, U5 and U2 showed the highest positive GCA effects, suggesting their strong ability to transmit favorable additive genetic effects to progeny. Conversely, parents with negative GCA effects exhibited unfavorable contributions to stem volume performance.

As shown in Table 3, 14 of the 40 controlled-pollinated families exhibit positive SCA values for stem volume, indicating the presence of non-additive genetic effects in these crosses. Among them, Families 21 (U × U), 15 (U × S), and 48 (U × G) show the highest SCA values (0.055, 0.029, and 0.020, respectively), ranking first to third across all combinations. Additional families, including 29, 8, 16, 12, 26, 27, 24, 46, 13, 17, and 28, also show positive SCA effects.

## 3. Discussion

### 3.1. Genetic Variation and Implications for Material Selection

Genetic variation in growth and stress resistance is controlled by multiple genes, with phenotypic performance shaped by genetic effects, environmental conditions, and genotype × environment interactions [22,23]. Substantial genetic variation provides the foundation for genetic improvement and sustained genetic gain [24]. In this study, the *E. urophylla* families and interspecific hybrid materials show significant differences in growth traits, survival rate, and cold tolerance, indicating considerable genetic variation within the test population and providing a strong basis for the selection of superior genotypes. The results from the 4-year-old and 6.5-year-old evaluations should, however, be interpreted in relation to stand age and the target rotation age. The 4-year-old evaluation mainly reflects early growth potential during the juvenile stage, whereas the 6.5-year-old evaluation represents a more advanced stage of plantation development and therefore provides greater information for assessing family-level mid-rotation volume production and sustained growth potential. As stand age increases, differences among families in growth performance and volume breeding values become more pronounced, and the rankings of some families change. These results indicate that evaluation at a single early age may overestimate the long-term genetic potential of some early fast-growing families, whereas dynamic evaluation across multiple stand ages is more effective for identifying materials with stable growth performance and sustained potential for genetic gain [25].

Among the 40 controlled-pollination families evaluated in this study, families 21 (U × U), 24 (U × G), and 48 (U × G) consistently show superior growth performance at both 4 and 6.5 years of age, indicating good growth stability and sustained production potential. Because the materials are still at a mid-rotation age, the 6.5-year evaluation provides more comprehensive information for long-term selection than the 4-year evaluation, but it does not fully replace assessments at mature ages or at the target rotation age. Therefore, future breeding programs should continue long-term evaluations through the target rotation age to further validate the genetic stability and final volume production potential of these superior families.

### 3.2. Heterosis and Its Genetic Regulation

Hybrid breeding is an important approach for genetic improvement in eucalyptus, as it enables the exploitation of heterosis through both additive and non-additive genetic effects, particularly dominance, to enhance overall progeny performance [26]. In this study, heterosis is evident at both 4 and 6.5 years of age and is more pronounced at 6.5 years, indicating that the hybrid advantage persists into the mid to late stages of stand development. Families 21, 48, and 24 maintain relatively high heterosis at both ages, whereas the open-pollinated family 38 shows volume performance comparable to, and in some cases higher than, that of the superior controlled-pollination combinations. This finding indicates that elite genotypes identified through natural selection also have considerable breeding value and can be used together with artificial hybridization to develop improved germplasm [27].

Further analyses of breeding values based on BLUP, general combining ability (GCA), and specific combining ability (SCA) indicate that individual-tree volume is influenced by both additive and non-additive genetic effects. The male parents DX8 (G) and KX1 (G) and the female parents U2 (U) and U5 (U) show relatively high GCA, indicating strong average genetic transmission ability and a relatively stable contribution of favorable additive effects to their progeny. These parents therefore have potential for recurrent selection and parental improvement in long-term breeding programs. Gustavo et al. [28] report that *Eucalyptus grandis*, *Eucalyptus camaldulensis*, and *Eucalyptus saligna* show relatively strong adaptation to cold conditions, providing a genetic basis for their use as parents in *Eucalyptus* breeding for cold-prone environments. This is generally consistent with the relatively high combining ability and cold tolerance observed for some of the corresponding parents in the present study. In contrast, combinations such as 21, 15, and 48 show relatively high SCA, indicating that non-additive genetic effects, including dominance and epistasis, contribute substantially to volume formation in specific hybrid combinations. Favorable genetic complementarity between parents is therefore an important factor underlying superior hybrid combinations. BLUP analysis further shows that families 38, 48, and 54 maintain relatively high volume breeding values across stand ages, indicating stable potential for genetic gain. These families are promising candidates for further genetic improvement, and subsequent evaluation of their clonal propagation capacity, clonal stability, and performance across environments would facilitate the conversion of heterosis from superior hybrid combinations into directly deployable clonal resources. This approach has important practical implications for heterosis breeding and the efficient utilization of genetic resources in eucalyptus.

### 3.3. Genetic Trade-Offs Between Growth and Cold Tolerance

Cold tolerance is an important factor limiting the deployment and sustainable development of eucalyptus plantations in cold marginal areas [29]. In this study, growth traits show an overall negative association with cold tolerance, with some high-volume families showing weaker cold tolerance, whereas strong cold-tolerant materials tend to have lower growth potential. This genetic trade-off may reflect resource allocation between rapid growth and stress defense and the differentiation of functional traits among genetic backgrounds [30]. However, not all fast-growing families show poor cold tolerance, and some materials achieve a favorable balance between productivity and cold adaptation. Families 46 (C × G), 23 (U × C), and 19 (U × C), for example, show both high volume and cold tolerance breeding values, indicating that growth and cold tolerance are not necessarily antagonistic. Appropriate parental combinations and multi-trait selection may therefore enable simultaneous improvement of productivity and environmental adaptation [31]. These families are promising candidates for eucalyptus breeding in cold marginal areas. Their genetic stability and environmental adaptability should be further validated through multi-site and multi-year trials extending toward the target rotation age, together with assessments of clonal propagation and field performance, to support the development and deployment of superior varieties and clones.

### 3.4. Genetic Dissection Based on Species and Cross-Combination Structure

Differences among BLUP, GCA, SCA, and heterosis estimates mainly arise from their representation of genetic effects at different levels. Integrating analyses from the family level to the species combination level provides a more comprehensive understanding of the genetic architecture of complex traits and can improve selection efficiency [32]. Previous studies by Xu et al. [3,33,34] show that *E*. *urophylla* families generally have strong and stable growth potential, combinations involving *E. grandis* tend to show higher volume growth, whereas those involving *E. camaldulensis* may have better cold adaptation. Our results are broadly consistent with these patterns. U × U combinations show relatively stable and sustained growth, whereas U × G combinations generally achieve higher volume gains, potentially due to genetic complementarity and heterosis. U × C and C × G combinations show relatively lower growth for some traits but stronger cold tolerance, suggesting that introducing more stress-adapted genetic backgrounds may improve environmental adaptation.

At the parental level, U-, G-, and C-derived parents such as U2, U5, DX8, KX1, and RT06 show high GCA, indicating broad and stable transmission of favorable genetic effects beyond specific crosses and highlighting their value for long-term genetic improvement. In contrast, combinations with high SCA indicate that favorable parental complementarity can generate performance beyond that expected from parental average effects. Thus, recurrent selection should prioritize parents with high GCA, whereas heterosis-based breeding should focus on specific combinations with high SCA and stable performance under target environments. Overall, integrating species background, GCA, SCA, heterosis, and BLUP breeding values supports a breeding strategy that progresses from superior parent selection and cross design to multi-age validation and clonal deployment, thereby improving the efficiency and precision of eucalyptus genetic improvement in cold environments.

## 4. Materials and Methods

### 4.1. Experimental Materials

The research base of the *E. urophylla* core breeding population (CATHs) was established in Xinhui District, Jiangmen City (22°33′08″ N, 113°03′06″ E). Superior families/individuals were selected as male parents and crossed with superior individuals, including selected female trees, in August 2004. Seeds from controlled crosses, open-pollinated seeds derived from seven superior female individuals, and control clones (three Rose gum clones and one WC3 clone) were collected in 2007. All seeds were sown and planted in October 2008. The parental materials and mating combinations are presented in Table 4.

### 4.2. Experimental Site and Experimental Design

The experiment was performed in Banling Village, Matou Town, Xinfeng City, Guangdong Province, China (24°10′26″ N, 114°16′13″ E). This region belongs to the subtropical maritime monsoon climate zone. The annual average temperature is 12.3 °C, with a monthly average low of 9.7 °C and an extreme minimum temperature of −5.8 °C. The annual precipitation is 1923 mm. The soil at the site was lateritic red soil with pH 3.6–4.0, soil organic matter content of 14.90 g kg^−1^, total N content of 0.96 g kg^−1^, total P content of 0.20 g kg^−1^, total K content of 35.20 g kg^−1^, hydrolytic N content of 34.25 mg kg^−1^, available P content of 0.64 mg kg−1, available K content of 18.56 mg kg−1, and available B content of 0.35 mg kg^−1^. A completely randomized block design was used to establish five blocks with 51 families and clones, and six replicates in 2006. Planting holes were 50 × 50 × 40 cm (length × width × height), and the planting space was 3 m × 2 m. A total of 0.75 kg of the special fertilizer for the eucalyptus plantation (NPK 8-15-8) was applied to each hole before planting, and the same fertilization usage (NPK 12-15-8) was applied. In January 2009, the extreme minimum temperature (−5.8 °C) appeared in the afforestation of Banling Village.

### 4.3. Data Collection and Measurements

The 4-year-old stage corresponds to the end of the rapid juvenile growth phase of *E. urophylla* plantations in South China, during which growth tends to stabilize. The 6.5-year-old stage is close to the typical economic rotation age (6–8 years) in subtropical regions, reflecting mid- to late-rotation growth performance. Field measurements were conducted in 2012 and 2014 for the 4-year-old and 6.5-year-old trials, respectively. Individual trees were used as the observational unit. Growth traits, cold tolerance, and survival rate were recorded for each family.

#### 4.3.1. Measurement of Growth Traits

Growth traits, including tree height (*H*), diameter at breast height (*DBH*), and individual stem volume (*SV*), were measured. Tree height (*H*, m) was measured using a hypsometer, and diameter at breast height (*DBH*, cm) was measured at 1.3 m above the ground using a diameter tape. For trees with branches, knots, or obvious stem deformities at the standard measurement height, DBH was measured at the nearest normal stem position above or below 1.3 m.

Individual stem volume (*SV*, m^3^·tree^−1^) was estimated using the empirical volume equation:

*SV* = (*H* × *DBH*^2^)/30000 (1)

where *H* is tree height (m), *DBH* is diameter at breast height (cm), and *SV* represents the stem volume of an individual tree (m^3^·tree^−1^).

#### 4.3.2. Assessment of Cold Tolerance

Cold tolerance (Cold-T) is evaluated following a winter cold event in 2014 using a five-point scale (1–5) based on the degree of damage. A score of 1 indicates complete lodging or uprooting; 2 indicates severe damage with more than half of the stem broken; 3 indicates moderate damage with stem inclination of 60–90°; 4 indicates slight damage with inclination of 30–60°; and 5 indicates no visible cold injury. Higher scores indicate greater cold tolerance.

#### 4.3.3. Survival Rate Measurement

Survival rate (*SR*) is calculated at the plot level based on the number of surviving trees in each plot:

*SR* = *Ns*/*Nt* (2)

where *Ns* is the number of surviving trees per plot, and *Nt* is the total number of planted trees per plot.

#### 4.3.4. Mean Annual Increment Calculation

Mean annual increment (*MAI*) was used to evaluate the average annual stem volume production per unit area of different genetic materials at different stand ages. Stand volume per hectare (*VP*, m^3^·ha^−1^) was first estimated from individual stem volume (*SV*), survival rate (*SR*), and the initial planting density. The initial spacing in the trial was 3 m × 2 m, corresponding to an area of 6 m^2^ per tree and an initial planting density of 10,000/6 trees ha^−1^. *VP* was calculated as:

*VP* = *SV* × *SR* × 10000/6 (3)

where *SV* is individual stem volume (m^3^·ree^−1^), *SR* is survival rate expressed as a proportion, 10,000 represents the area of one hectare (m^2^), and 6 represents the initial area occupied by each tree (m^2^·tree^−1^). *MAI* (m^3^·ha^−1^·yr^−1^) was subsequently calculated as:

*MAI* = *VP*/*Age* (4)

where *VP* is stand volume per hectare (m^3^·ha^−1^) and *Age* is stand age (yr). Thus, *MAI* represents the average annual accumulation of stand volume per unit area over the corresponding rotation period.

#### 4.3.5. Calculation of Genetic Parameters

Based on the growth trait measurement results, genetic parameters including family volume breeding value (*BV*), general combining ability (*GCA*), specific combining ability (*SCA*), and heterosis were further calculated. Among these, family breeding value was used to evaluate the genetic potential of each family and to screen genetic materials with outstanding overall performance; heterosis analysis was used to assess the degree of gain of hybrid offspring relative to the control clones, open-pollinated families, and the overall mean; *GCA* and *SCA* reflected the average combining ability of parents and the combining ability of specific cross combinations, respectively. The calculation methods for the above genetic parameters are described in Section 4.4.

### 4.4. Data Analysis

Data organization and statistical analyses are performed using Microsoft Excel 2021 (Microsoft Corp., Redmond, WA, USA), SPSS 26.0, and SAS software 9.4 (SAS Institute Inc., Cary, NC, USA). The experiment is arranged in a randomized complete block design (RCBD), with individual trees treated as the experimental unit. All observations are assumed to be independent.

#### 4.4.1. Assumption Checking for Continuous Traits

Assumption checks for continuous traits (*H*, *DBH*, *SV*, and *MAI*) are performed using the Shapiro–Wilk test and Q-Q plots for normality, and Levene’s test for homogeneity of variance. Analysis of variance (ANOVA) is conducted when assumptions are satisfied.

#### 4.4.2. Analysis of Variance for Continuous Traits

Continuous traits, including tree height (*H*), diameter at breast height (*DBH*), and stem volume per tree (*SV*), were analyzed using a mixed linear model, with family specified as a fixed effect and block and family × block interaction included as random effects to account for environmental heterogeneity among experimental plots. The statistical model was as follows:

*Y_ijk_* = *μ* + *B_i_* + *G_j_* + (*BG*)*_ij_* + *e_ijk_* (5)

where *Y_ijk_* is the observation of the *k*th individual of the *j*th family in the *i*th block; *μ* is the overall mean; *B_i_* is the random effect of the *i*th block; *G_j_* is the fixed effect of the *j*th family; (*BG*)*_ij_* is the random family × block interaction; and *e_ijk_* is the random residual error. Restricted maximum likelihood (REML) was used to estimate the variance components of the random effects, and the significance of the fixed family effect was assessed using an F-test. Multiple comparisons among family means were performed using Tukey’s HSD test, with statistical significance set at *p* < 0.05.

#### 4.4.3. Analysis of Cold Tolerance

Cold tolerance (*Cold-T*) is assessed using an ordinal scale ranging from 0 to 5. As an ordered categorical variable, *Cold-T* does not meet the distributional assumptions required for parametric analysis. Therefore, differences among families are evaluated using the Kruskal–Wallis nonparametric test. Results are presented as boxplots, with statistical significance defined at *p* < 0.05.

#### 4.4.4. Analysis of Survival Rate

Survival rate (*SR*) is a proportional variable calculated as the ratio of surviving trees to the total number of trees in each plot. To improve normality and homogeneity of variance, *SR* is transformed prior to statistical analysis using the arcsine square-root transformation:

(6) $$y\;=\;arcsin(\sqrt{P})$$

where *P* denotes survival rate (0–1). The transformed data are analyzed using analysis of variance (ANOVA) to test differences among families, followed by Tukey’s HSD test for multiple comparisons. Results are presented as boxplots, with statistical significance defined at *p* < 0.05.

#### 4.4.5. Analysis of Breeding Values

To evaluate the genetic contribution of parents to progeny stem volume, breeding values (*BV*) are estimated using the best linear unbiased prediction (BLUP) method. The model is expressed as follows:

*BV* = *E*(*g*) + {*Cov*(*g*, *y*)/*Var*(*y*)} (*y* − *a*) (7)

where *BV* is the predicted breeding value; (*E*(*g*)) is the mean genetic value of all families; (*Cov*(*g*, *y*)) is the covariance between the observed value (*y*) and the genetic value (*g*); (*Var*(*y*)) is the variance of the observed value; and (*a*) is the expected value of the observed trait (*y*), i.e., the population mean of the trait. Because the predicted breeding values are expressed relative to the population mean genetic level, positive and negative *BV* estimates indicate genetic performance above and below the population mean, respectively, and do not imply that the corresponding phenotypic trait values are negative or positive.

#### 4.4.6. Heterosis

Using individual stem volume as the evaluation trait, heterosis in hybrid families is analyzed. *H_op_* (over-population heterosis) is defined as the relative advantage of family stem volume compared with the mean of all 51 tested materials; *H_e_* (over-standard heterosis) is defined as the relative advantage compared with the best hybrid family (No. 38 at age 4 and No. 21 at age 6.5); *H_ck_* (over-clonal heterosis) is defined as the relative advantage compared with the mean of four clonal controls (Nos. 52–55); *H_m_* (over-natural open-pollinated heterosis) is defined as the relative advantage compared with the mean of seven open-pollinated families; and *H_f_* (over-controlled open-pollinated heterosis) is defined as the relative advantage compared with the mean of all controlled-pollinated families. Each heterosis index is calculated as follows [18].

*H_op_* = (*F*_1_ − *MP*)(*MP*)·100 (8)

*H_e_* = (*F*_1_ − *CK*_1_)(*CK*_1_)·100 (9)

*H_m_* = (*F*_1_ − *OP*)(*OP*)·100 (10)

*H_ck_* = (*F*_1_ − *CK*_2_)(*CK*_2_)·100 (11)

*H_f_* = (*F*_1_ − *CP*)(*CP*)·100 (12)

where (*F*_1_) denotes the mean individual stem volume of hybrid families; (*MP*) denotes the population mean; (*CK*_1_) denotes the mean value of superior control families; (*OP*) denotes the mean value of open-pollinated families; (*CK*_2_) denotes the mean value of clonal controls; and (*CP*) denotes the mean value of controlled-pollinated families.

#### 4.4.7. Combining Ability

To evaluate the genetic effects of parents and the combining ability of hybrid combinations, a mixed linear model based on an irregular diallel mating design is used for combining ability analysis. The statistical model is specified as follows:

*Y_ijkl_* = *μ* + *M_i_* + *F_j_* + (*FM*)*_ij_* + *B_k_* + *e_ijkl_* (13)

where *Y_i__j__kl_* is the observed value of the lth individual from the cross between female parent i and male parent *j* in block *k*; *μ* is the overall mean; *M_i_* and *F_j_* represent the general combining ability (GCA) effects of the female and male parents, respectively; *(FM)_ij_* denotes the specific combining ability (SCA) effect; *B_k_* is the block effect; and *e_ijkl_* is the random residual error. Because the objective of this study was to estimate the genetic variation among parents and hybrid combinations within the target breeding population, maternal GCA, paternal GCA, and SCA were all treated as random effects. Blocks were included primarily to account for spatial environmental heterogeneity within the experimental site rather than to compare specific blocks; therefore, block effects were also treated as random effects to separate block-associated environmental variation from genetic effects. Restricted maximum likelihood (REML) was used to estimate the variance components of the random effects, and the predicted effects of GCA and SCA were subsequently obtained.

## 5. Conclusions

Based on 4- and 6.5-year-old experimental materials comprising *E*. *urophylla* and interspecific hybrids, this study systematically evaluated growth traits, cold tolerance, and their genetic basis. Substantial genetic variation was observed among the evaluated materials. Comprehensive evaluation across the two stand ages identified fast-growing families, including 38 (UN), 21 (U × U), and 48 (U × G), as well as families combining superior growth and cold tolerance, including 19 (U × C), 21 (U × U), 23 (U × C), 24 (U × G), and 29 (C × G). Although breeding values for stem volume and cold tolerance showed an overall negative trend, families 46 (U × G), 19 (U × C), 23 (U × C), and 29 (C × G) exhibited high breeding values for both traits, indicating strong overall genetic potential.

Heterosis analysis showed that stem volume heterosis was more pronounced at later stand ages. Families 21 (U × U), 48 (U × G), and 24 (U × G) maintained relatively stable heterosis across stand ages, while open-pollinated family 38 (UN) also showed superior stem volume performance. Combining ability analysis identified male parents DX8 (G) and KX1 (G) and female parents U5 and U2 as promising candidates for future hybrid breeding. Among the tested species combinations, *E. urophylla* × *E. grandis*, *E. urophylla* × *E. camaldulensis*, and *E. camaldulensis* × *E. grandis* showed relatively high growth potential and cold tolerance adaptation.

Overall, this study identified families and parental materials with high breeding potential based on growth performance, cold tolerance, breeding values, heterosis, and combining ability, providing a basis for cold-tolerant *E. urophylla* breeding, parental selection, and efficient utilization of heterosis.

## Figures and Tables

**Table 1:** Tests of fixed effects on growth traits among different genetic materials at 4 and 6.5 years.

Age	Genetic Material	N	H		DBH		SV	
			F	*p*	F	*p*	F	*p*
4 years	CCF	762	4.935	<0.0001	5.541	<0.001	5.894	<0.001
	OPF	152	6.338	<0.001	5.062	<0.001	7.550	<0.001
	CL	98	2.383	0.0761	3.006	0.0356	4.213	0.0083
6.5 years	CCF	688	2.848	0.0019	2.580	0.0047	2.335	0.0107
	OPF	133	8.158	<0.001	6.865	<0.001	7.160	<0.001
	CL	92	2.956	0.0384	3.351	0.0238	3.135	0.0309

Note: CCF, controlled-pollinated families; OPF, open-pollinated families; CL, clonal controls. F and *p* values represent tests of the fixed family effect. Random effects (block and family × block interaction) were estimated using REML and are not presented as F-tests in this table.

**Table 2:** General combining ability (GCA) effects of superior and inferior parents for volume growth at 6.5 years old.

Parent Type	Parent	GCA Estimate (ĝi)	Relative GCA (%)
Female	DX8	0.043	65.678
Female	KX1	0.025	38.144
Female	T2	−0.022	−33.793
Female	RT02	−0.044	−66.049
Male	U5	0.025	38.144
Male	U2	0.014	20.945
Male	U20	−0.019	−28.726
Male	U56	−0.020	−30.179

Note: Only parents showing the two highest positive and two lowest negative GCA effects are presented. The complete GCA estimates for all parents are provided in Supplementary Table S2.

**Table 3:** Specific combining ability (SCA) effects and relative SCA effects of hybrid combinations for individual volume at 6.5 years old.

No.	Hybrid Combination	SCA Effect Estimate (S^ij)	Relative SCA Effect (%)	No.	Hybrid Combination	SCA Effect Estimate (S^ij)	Relative SCA Effect (%)
21	U15 × ZU6	0.055	83.571	43	U1 × T4	−0.001	−1.663
15	U22 × s1	0.029	44.111	44	U1 × T5	−0.001	−1.663
48	U1 × DX8	0.020	30.179	45	U1 × KX3	−0.001	−1.663
29	RT10 × FS1	0.019	29.526	47	U1 × KX11	−0.001	−1.663
8	U2 × D4	0.017	25.501	25	U56 × RT07	−0.001	−1.663
16	U22 × D4	0.016	24.777	49	U1 × DX25	−0.001	−1.663
12	U20 × D4	0.015	22.615	51	U1 × TH9512	−0.001	−1.663
26	RT10 × 507	0.012	18.812	6	U2 × b1	−0.002	−3.396
27	RT10 × 510	0.012	18.812	2	UT8 × s1	−0.005	−7.675
24	U5 × KX1	0.012	18.812	1	UT8 × b1	−0.007	−10.086
46	U1 × KX9	0.012	18.423	18	U15 × RT05	−0.007	−10.204
13	U20 × D5	0.010	15.416	19	U15 × RT09	−0.007	−10.204
17	U22 × D5	0.003	4.307	20	U15 × W5	−0.007	−10.204
28	RT10 × KX9	0.001	1.237	22	U15 × EX	−0.007	−10.204
5	T8 × D6	0.000	−0.337	50	U1 × FS1	−0.007	−11.038
9	U2 × D5	−0.001	−1.288	31	U1 × ZU6	−0.011	−16.469
30	U1 × U20	−0.001	−1.663	4	UT8 × D5	−0.012	−17.973
32	U1 × LT4	−0.001	−1.663	23	U2 × RT06	−0.014	−20.945
41	U1 × T2	−0.001	−1.663	33	RT10 × RT02	−0.025	−38.144
42	U1 × T3	−0.001	−1.663	3	UT8 × D4	−0.034	−51.898

**Table 4:** The parents’ information of the families in trials.

No.	Parents	♀ Species	♂ Species	No.	Parents	♀ Species	♂ Species	No.	Parents	♀ Species	♂ Species
1	UT8 × b1	U	B	22	U15 × EX	U	U	39	U56 (F)	U	UN
2	UT8 × s1	U	S	23	U2 × RT06	U	C	40	UT8 (F)	U	UN
3	UT8 × D4	U	D	24	U5 × KX1	U	G	41	U1 × T2	U	T
4	UT8 × D5	U	D	25	U56 × RT07	U	C	42	U1 × T3	U	T
5	UT8 × D6	U	D	26	RT10 × 507	C	P	43	U1 × T4	U	T
6	U2 × b1	U	B	27	RT10 × 510	C	P	44	U1 × T5	U	T
8	U2 × D4	U	D	28	RT10 × KX9	C	G	45	U1 × KX3	U	G
9	U2 × D5	U	D	29	RT10 × FS1	C	G	46	U1 × KX9	U	G
12	U20 × D4	U	D	30	U1 × U20	U	U	47	U1 × KX11	U	G
13	U20 × D5	U	D	31	U1 × ZU6	U	U	48	U1 × DX8	U	G
15	U22 × s1	U	S	32	U1 × LT4	U	T	49	U1 × DX25	U	G
16	U22 × D4	U	D	33	RT10 × RT02	C	C	50	U1 × FS1	U	G
17	U22 × D5	U	D	34	U2 (F)	U	UN	51	U1 × TH9512	U	C
18	U15 × RT05	U	C	35	U5 (F)	U	UN	52	Clone/G2 (O)	-	-
19	U15 × RT09	U	C	36	U15 (F)	U	UN	53	Clone/G3 (O)	-	-
20	U15 × W5	U	A	37	U20 (F)	U	UN	54	Clone/G4 (O)	-	-
21	U15 × ZU6	U	U	38	U22 (F)	U	UN	55	Clone/WC3 (O)	-	-

Note: U: *E. urophylla* family, UN: the family of source unknown, F: female parent, O: control clones. The lettres U, G, T, C, S, D, B, A and P represent *Eucalyptus urophylla*, *Eucalyptus grandis*, *Eucalyptus tereticornis*, *Eucalyptus camaldulensis*, *Eucalyptus saligna*, *Eucalyptus dunnii*, *Eucalyptus benthamii*, *Eucalyptus ABL No.12* and *Eucalyptus pellita*, respectively. Controlled-pollinated families (1–33, 41–51); Open-pollinated families (34–40); clonal controls (52–55).

## Data Availability

All relevant data are within the manuscript and its Supporting Information files.

## Supplementary Materials

The following supporting information can be downloaded at: https://www.mdpi.com/article/10.3390/plants15162505/s1, File S1: Original datasets for two study years. Table S1. Predicted breeding value (BV) of the hybrid families with BLUP method. Table S2. General combining ability effects and relative GCA effects for individual volume of parental materials at 6.5 years old.

## Abbreviations

The following abbreviations are used in this manuscript:

GCA	General Combining Ability
SCA	Special Combining Ability
*BV*	Breeding values
BLUP	Best Linear Unbiased Prediction
CCF	Controlled-pollinated families
OPF	Open-pollinated families
CL	Clonal controls
*SV*	Stem Volume
*H*	Tree Height
*DBH*	Diameter at Breast Height
*MAI*	Mean Annual Increment
*Cold-T*	Cold Tolerance
*SR*	Survival rate
*H_op_*	Over-population heterosis
*He*	Over-standard heterosis
*H_ck_*	Over-clone heterosis
*H_m_*	Over-natural open-pollinated heterosis
*H_f_*	Over-controlled open-pollinated heterosis
U	*Eucalyptus urophylla*
G	*Eucalyptus grandis*
T	*Eucalyptus tereticornis*
C	*Eucalyptus camaldulensis*
S	*Eucalyptus saligna*
D	*Eucalyptus dunnii*
B	*Eucalyptus benthamii*
A	*Eucalyptus ABL No.12*
P	*Eucalyptus pellita*
UN	Unknown
F	Female parent
O	Control clone
