# Supplementary material for: Genetic Evaluation and Selection of Eucalyptus urophylla Families and Hybrids for Cold Tolerance in Northern Guangdong, South China
Source: Plants (Basel). 2026 Aug 19;15(16):2505. doi: 10.3390/plants15162505 (PMC13516064; doi:10.3390/plants15162505)
Supplement: Supplementary file 1 [file plants-15-02505-s001.zip › Table S1.pdf]

**Table S1.** Predicted breeding value（BV）of the hybrid families with BLUP method.

| The breeding value of 4 a | | | | | The breeding value of 6.5 a | | | | | |
|---|---|---|---|---|---|---|---|---|---|---|
| **No.** | **SV** | **H** | **DBH** | **MAI** | **No.** | **SV** | **H** | **DBH** | **MAI** | ***Cold-R*** |
| 54 | 0.0302 | 2.3398 | 3.7737 | 10.1056 | 38 | 0.0542 | 1.0173 | 2.3970 | 7.5712 | -0.2208 |
| 38 | 0.0300 | 2.4797 | 3.6924 | 12.3728 | 48 | 0.0434 | -0.5301 | 1.1216 | -2.6296 | -0.0972 |
| 52 | 0.0188 | 1.3075 | 2.6318 | 6.1350 | 54 | 0.0330 | 0.3991 | 2.1193 | 3.7524 | -0.1637 |
| 24 | 0.0182 | 0.7452 | 2.1250 | 3.9719 | 24 | 0.0274 | -1.2834 | 0.4346 | 4.3856 | -0.1922 |
| 48 | 0.0177 | 1.3777 | 2.7311 | 5.9690 | 53 | 0.0269 | -1.5313 | 0.0994 | -3.5168 | -0.2465 |
| 47 | 0.0130 | 1.1556 | 2.5217 | 7.2470 | 29 | 0.0212 | -1.0661 | 0.5920 | -2.4717 | 0.0005 |
| 29 | 0.0105 | 0.8799 | 2.2206 | 3.6971 | 23 | 0.0185 | -1.7336 | -0.0891 | 18.1381 | 0.0495 |
| 19 | 0.0104 | 1.0953 | 2.3646 | 6.1522 | 42 | 0.0183 | -1.5229 | 0.1596 | 6.8172 | -0.0429 |
| 53 | 0.0090 | 0.4089 | 1.7132 | 3.3695 | 46 | 0.0177 | -1.5628 | 0.0852 | 5.1785 | 0.2556 |
| 26 | 0.0084 | 0.7034 | 1.2358 | 3.2016 | 21 | 0.0158 | -0.0763 | 0.3095 | -0.5709 | -0.0198 |
| 5 | 0.0071 | 0.5464 | 1.7322 | 0.5496 | 19 | 0.0154 | -0.6984 | 0.8502 | 2.1719 | 0.1199 |
| 46 | 0.0058 | 0.3991 | 1.7159 | 3.0058 | 6 | 0.0120 | -2.2497 | -1.1200 | -3.5865 | 0.1308 |
| 50 | 0.0056 | 0.0741 | 1.4340 | 5.8693 | 5 | 0.0119 | -1.1808 | 0.2841 | -2.6981 | -0.2476 |
| 28 | 0.0051 | 0.7368 | 2.0825 | 0.5822 | 47 | 0.0110 | -0.8024 | 0.8781 | 11.1074 | -0.1759 |
| 49 | 0.0049 | 0.4168 | 1.7215 | 3.0131 | 50 | 0.0085 | -1.9924 | -0.2729 | 1.2384 | -0.2709 |
| 21 | 0.0043 | 0.3404 | 0.6822 | 0.3144 | 20 | 0.0077 | -1.8658 | -0.2067 | 6.4277 | -0.1325 |
| 20 | 0.0034 | 0.1253 | 1.4413 | 4.8139 | 35 | 0.0054 | -2.1423 | -0.6509 | -4.5069 | -0.0429 |
| 31 | 0.0033 | 0.2701 | 1.3770 | 1.7560 | 26 | 0.0052 | 0.0811 | 0.6737 | 2.7512 | -0.0760 |
| 42 | 0.0031 | 0.4767 | 1.8216 | 0.6997 | 30 | 0.0047 | -3.0457 | -1.4659 | -0.2563 | 0.2868 |
| 43 | 0.0025 | 0.3850 | 1.6604 | 3.3047 | 52 | 0.0042 | -0.5560 | 1.0580 | 7.2344 | -0.3268 |
| 23 | 0.0025 | 0.2337 | 1.5422 | 3.9426 | 43 | 0.0036 | -1.5107 | 0.0825 | 5.2779 | 0.1258 |
| 1 | 0.0012 | -0.2366 | 0.7880 | -3.6316 | 15 | 0.0035 | -0.2657 | 0.2152 | -7.1365 | 0.0434 |
| 15 | 0.0001 | 0.2775 | 0.6900 | -5.3709 | 9 | 0.0025 | -2.9717 | -1.4762 | -5.3358 | 0.2461 |
| 8 | 0.0000 | 0.2187 | 0.4578 | -2.2991 | 8 | 0.0020 | -0.0742 | 0.1957 | -2.5582 | 0.0058 |
| 35 | -0.0007 | -0.3227 | 0.8487 | -0.9894 | 1 | 0.0003 | -1.8224 | -0.5240 | -6.0497 | 0.0683 |
| 4 | -0.0007 | 0.0853 | 0.3067 | -8.8027 | 22 | -0.0028 | -0.7449 | -0.3941 | -1.9805 | -0.0198 |
| 2 | -0.0008 | -0.3908 | 0.5371 | -6.5567 | 4 | -0.0030 | -0.2189 | 0.0430 | -2.9055 | 0.0058 |
| 45 | -0.0011 | 0.2121 | 1.5322 | -0.8580 | 49 | -0.0054 | -1.5231 | 0.1078 | 5.5695 | -0.1387 |
| 22 | -0.0011 | -0.2780 | -0.0140 | -5.6317 | 2 | -0.0059 | -1.8559 | -0.6711 | -7.0526 | 0.0028 |
| 18 | -0.0015 | 0.0122 | 1.3226 | -0.3084 | 16 | -0.0071 | -1.4260 | -0.6596 | -4.4872 | 0.0544 |
| 13 | -0.0016 | 0.1950 | 0.7944 | 0.2988 | 13 | -0.0072 | -0.6582 | 0.0668 | -1.1868 | 0.0694 |
| 51 | -0.0023 | 0.4077 | 1.6696 | -1.7052 | 18 | -0.0074 | -1.9826 | -0.3259 | -1.1658 | 0.1498 |
| 37 | -0.0026 | -0.4180 | 0.7658 | -3.9908 | 28 | -0.0076 | -1.2347 | 0.4371 | 1.2298 | -0.2248 |
| 6 | -0.0030 | -0.8111 | 0.0517 | -2.9526 | 31 | -0.0078 | -1.3684 | 0.0073 | 0.7231 | 0.0042 |
| 55 | -0.0038 | 0.2211 | 1.5539 | -0.5697 | 3 | -0.0092 | -0.9634 | -0.7426 | -2.4988 | 0.0569 |
| 44 | -0.0040 | 0.6115 | 1.9742 | -0.2022 | 51 | -0.0104 | -1.4631 | 0.1109 | -2.3876 | -0.0687 |
| 16 | -0.0051 | -0.4459 | 0.1442 | -5.0486 | 45 | -0.0115 | -1.7762 | -0.1154 | -3.5288 | -0.2085 |
| 3 | -0.0056 | -0.6006 | -0.4703 | -3.7641 | 12 | -0.0116 | -2.2019 | -1.2167 | -2.7339 | -0.0399 |
| 41 | -0.0063 | -0.4010 | 0.8479 | -3.7620 | 32 | -0.0130 | -2.4255 | -0.8730 | -0.3884 | -0.0131 |
| 17 | -0.0064 | -0.3250 | 0.9279 | -5.3094 | 17 | -0.0134 | -2.2680 | -0.6716 | -4.8141 | 0.1023 |

| | | | | | | | | | | |
|---|---|---|---|---|---|---|---|---|---|---|
| 32 | -0.0069 | -0.5216 | 0.6901 | -1.8406 | 37 | -0.0150 | -2.2680 | -0.7561 | -5.8369 | 0.2054 |
| 9 | -0.0076 | -1.0964 | 0.0491 | -3.0436 | 36 | -0.0156 | -3.0367 | -1.4190 | -1.6909 | 0.2081 |
| 40 | -0.0101 | -0.8565 | 0.0972 | -1.5946 | 34 | -0.0162 | -2.8499 | -1.2860 | -5.5349 | 0.1932 |
| 30 | -0.0103 | -1.0770 | 0.1369 | -1.8579 | 40 | -0.0166 | -2.4309 | -1.1858 | 3.0490 | -0.0464 |
| 34 | -0.0115 | -0.9091 | 0.2979 | -3.2364 | 55 | -0.0166 | -1.7864 | -0.1092 | 4.6396 | -0.0755 |
| 12 | -0.0115 | -0.9181 | -0.1785 | -6.0794 | 27 | -0.0192 | -4.1011 | -2.5241 | -4.2753 | 0.3031 |
| 36 | -0.0132 | -1.0281 | 0.2178 | -0.8197 | 44 | -0.0199 | -1.4028 | 0.2977 | 9.1116 | 0.2556 |
| 25 | -0.0146 | -1.0442 | 0.2533 | -1.9095 | 39 | -0.0204 | -4.4213 | -2.8510 | -0.0744 | 0.1308 |
| 39 | -0.0191 | -2.3876 | -1.2250 | -2.3944 | 25 | -0.0249 | -3.1272 | -1.4442 | 4.9656 | 0.0711 |
| 27 | -0.0205 | -2.0777 | -0.8994 | -4.1064 | 41 | -0.0252 | -2.3466 | -0.7519 | 0.0269 | 0.0222 |
| 33 | -0.0242 | -2.3741 | -1.1121 | -7.4300 | 33 | -0.0481 | -4.5291 | -2.8417 | 7.7988 | 0.0521 |
