# Supplementary material for: Genetic Evaluation and Selection of Eucalyptus urophylla Families and Hybrids for Cold Tolerance in Northern Guangdong, South China
Source: Plants (Basel). 2026 Aug 19;15(16):2505. doi: 10.3390/plants15162505 (PMC13516064; doi:10.3390/plants15162505)
Supplement: Supplementary file 1 [file plants-15-02505-s001.zip › Table S2.pdf]

Table S2. General combining ability effects and relative GCA effects for individual volume of parental materials at 6.5 years old

Parent type	Parent	GCA estimate (ĝi)	Relative GCA (%)
Female	DX8	0.043	65.678
Female	KX1	0.025	38.144
Female	RT06	0.017	26.192
Female	T3	0.017	25.473
Female	RT09	0.015	23.368
Female	FS1	0.014	21.291
Female	507	0.014	20.477
Female	D6	0.013	18.945
Female	KX11	0.010	14.416
Female	b1	0.007	10.562
Female	W5	0.007	10.543
Female	U20	0.004	6.780
Female	KX9	0.004	6.561
Female	T4	0.004	5.640
Female	ZU6	0.001	2.261
Female	KX1	0.025	38.144
Female	s1	-0.004	-5.627
Female	DX25	-0.006	-8.358
Female	D5	-0.006	-8.709
Female	RT05	-0.007	-10.926
Female	TH9512	-0.010	-15.860
Female	KX3	-0.010	-15.901
Female	EX	-0.011	-17.384
Female	LT4	-0.013	-19.052
Female	T5	-0.017	-26.264
Female	D4	-0.019	-29.323
Female	510	-0.020	-29.943
Female	RT07	-0.020	-30.179
Female	T2	-0.022	-33.793
Female	RT02	-0.044	-66.049
Male	U5	0.025	38.144
Male	U2	0.014	20.945
Male	U15	0.007	10.204
Male	U1	0.001	1.663
Male	T8	0.0002	0.337
Male	U22	-0.011	-17.205
Male	RT10	-0.012	-18.812
Male	U20	-0.019	-28.726
Male	U56	-0.020	-30.179
